# Evaluating the systematic implementation of the ‘Let Me Decide’ advance care planning programme in long term care through focus groups: staff perspectives

**DOI:** 10.1186/s12904-015-0051-x

**Published:** 2015-11-03

**Authors:** Nicola Cornally, Ciara McGlade, Elizabeth Weathers, Edel Daly, Carol Fitzgerald, Rónán O’Caoimh, Alice Coffey, D. William Molloy

**Affiliations:** Centre for Gerontology and Rehabilitation, University College Cork, St Finbarrs’ Hospital, Cork City, Ireland; Health Service Executive, Mallow General Hospital, Mallow, Cork Ireland; Catherine McAuley School of Nursing and Midwifery, University College Cork, Cork, Ireland; Health Research Board Clinical Research Facility Galway, National University of Ireland, Geata an Eolais, University Road, Galway, Ireland

**Keywords:** Advance care planning, Long-term care, Qualitative, Focus groups, Challenges, Barriers, Benefits

## Abstract

**Background:**

The ‘Let Me Decide’ Advance Care Planning (LMD-ACP) programme offers a structured approach to End-of-Life (EoL) care planning in long-term care for residents with and without capacity to complete an advance care directive/plan. The programme was implemented in three homes in the South of Ireland, with a view to improving quality of care at end of life. This paper will present an evaluation of the systematic implementation of the LMD-ACP programme in the homes.

**Methods:**

Focus groups were conducted with 15 Clinical Nurse Managers and two Directors of Nursing where the programme had been implemented. A semi-structured topic guide was used to direct questions that addressed implementation process, challenges implementing advance care planning, advantages/disadvantages and recommendations for the future. Data was analysed using manifest content analysis.

**Results:**

Five key categories emerged, with 16 corresponding subcategories. These subcategories emerged as a result of 37 codes. Key benefits of the programme included enhancing communication, changing the care culture, promoting preference-based care and avoiding crisis decision making. Establishing capacity among residents and indecision were among the main challenges reported by staff.

**Discussion:**

A number of recommendations were proposed by participants and included multi-disciplinary team involvement, and a blended approach to education on the topic. According to participants relationships with residents deepened, there was a more open and honest environment with family, end of life care focused more on symptom management, comfort and addressing spiritual care needs as opposed to crisis decision making and family conflict.

**Conclusion:**

The introduction of the LMD-ACP programme enhanced the delivery of care in the long-term care sites and led to a more open and positive care environment.

## Background

Advance care planning (ACP) is a process of communication between an individual, their healthcare providers, and often those close to them about their values and preferences for their future treatment and care. The primary goal of ACP is to help people document their wishes about what life-sustaining treatments they would or would not wish to receive in the future, in the event that they lose the capacity to make, or communicate, these decisions. One potential output of ACP is an Advance Care Directive (ACD).

An ACD is a record (usually written) of an informed decision, made by a person with decision-making capacity; regarding medical treatment they would wish to receive (or not receive) should they subsequently lose capacity. An ACD is only valid if it is made voluntary, by a competent informed person and should only be used or acted upon if the person becomes incompetent to make medical decisions. ACDs can improve satisfaction with end-of-life care and facilitate choice regarding place of death [1]. The Council of Europe has promoted the use of ACDs in EU member states to enhance self-determination among citizens [2]. According to the World Health Organisation [3] ACDs are “a mechanism by which a competent individual expresses his or her wishes should circumstances arise in which he or she no longer is able to make rational and sound decisions regarding his or her medical treatment.”

One of the few randomised controlled trials [4] of ACDs in LTC, conducted in Canada, found that the majority of residents completing directives chose to remain in the LTC facility and receive palliative care. The study also demonstrated a statistically significant reduction in health service utilisation (*p* < 0.003) without a compromise in family satisfaction with care. A recent review of RCTs conducted with older adults [5] highlighted the need for further research examining the impact of ACP interventions on quality of end-of-life care, and the quality of the death and dying experience.

The current research, on which this paper is based, was a before-after, two year study to examine the effect of systematically implementing an advance care planning programme and palliative care education intervention in three nursing homes in Ireland, using ‘Let Me Decide’ [6]. The primary outcome of this study is quality of care at end of life, and the quality of the dying and death experience from family and staff perspectives. The ‘Let Me Decide’ Advance Care Planning (LMD-ACP) programme offers a structured approach to EoL care planning in the LTC setting for both residents with and without capacity to complete an advance care directive/plan. The programme involves educating staff on advance care planning, completing an advance care directive and palliative care approaches at end of life. Structured advance care directives and end of life decision care plans were provided, along with education material for families and residents. Part of the advance care planning process includes capacity assessment using the standardised mini mental state examination (SMMSE). If a competent resident wishes to complete an ACD they are educated on treatment decisions. Prior to completing the form they are assessed using the Screening Instrument to Assess Competency to Complete an Advance Directive (SIACAD). This quality assurance measure is unique to the LMD-ACD programme [7] and measures capacity to understand the details surrounding end of life decision making.

The programme was initially designed for use in Canada and was successfully implemented in LTC in several studies [4]. In Australia, a controlled study that utilised the LMD-ACD found that ACP, alongside implementation of a hospital-in-the-home scheme, can result in decreased hospital admission and mortality of nursing home residents [8]. In a recent review of published studies on ACP programmes in LTC homes, the LMD programme was classed as dementia-friendly based on a set of criteria in the Dementia Policy Lens Toolkit [9].

The larger study on which this paper is based examined both quantitative and qualitative methods. Quantitatively the effectiveness of LMD programme is measured from an economics perspective and impact on quality of care, nurses knowledge, ACD/ACP uptake rates, compliance with resident’s wishes at end of life and barriers to implementing the programme. Qualitative evaluation seeks to gain a deeper understanding, from staff involved with the process, of the impact of the programme from initial introduction to embedding into everyday practice.

This paper reports the qualitative evaluation of the systematic implementation of the ‘Let Me Decide’ advance care directive and palliative care education programme in three long-term care sites in Ireland, from the staff perspective participating in focus groups i.e. staff nurses/managers in the nursing homes. The impact that the programme had on practice was collected using field notes, results which fall outside the scope of this paper and are the subject of a forthcoming publication [10].

## Methods

A qualitative descriptive approach was applied to this phase of evaluation. Ethical approval for the overall study was granted by the Clinical Research Ethics Committee of the Cork Teaching Hospitals. Data was collected using focus groups. These were conducted with staff working in the three long-term care sites where the programme was implemented. Staff were invited to participate via email correspondence. All staff involved in either delivering ACP or overseeing the implementation of the programme at senior management level, were invited to participate in the focus groups. Three separate focus groups were conducted with samples ranging from two in the smallest home to seven and eight, respectively in the larger sites. Focus groups were chosen over individual interviews as most of the training and feedback site visits were conducted in groups and this appeared to be the environment in which participants were most comfortable to explore and discuss any issues relating to end of life care planning. A great sense of comradery and interaction was noted between the key informant’s right throughout the programme implementation and the researchers agreed that discussions and subsequent data would be far richer in a group setting. The smallest home had a bed capacity of 79, while the other two homes had 97 and 120 beds, respectively. These homes are reflective of the average nursing home occupancy range in Ireland. The overall sample for analysis consisted of 15 Clinical Nurse Managers and two Directors of Nursing (*n* = 17).

### Focus groups

A semi-structured topic guide was developed and contained a number of open ended questions. The focus groups began with general introductions then proceeded to opening questions, introductory questions, transition questions and were closed with a number of summary ending questions. Questions addressed the implementation process, challenges implementing advance care planning, advantages/disadvantages and recommendations for the future. Written consent was gained prior to conducting the focus groups. Each session was digitally recorded. The length of the focus groups varied from 51 min to 1 h 12 min.

### Data analysis

Data from the focus groups were first transcribed to a Word document. Subsequently the data were analysed using manifest content analysis [11], whereby the ‘obvious components’ of the text were highlighted using coloured markers and grouped according to the predetermined categories of the interview guide. These sections of text or statements were subsequently labelled ‘meaning units’. The meaning units were then reread and the key words extracted to form ‘condensed meaning units’. These data were then presented in table format for ease of interpretation and further commonalities were assembled. Codes were then developed to reflect the meaning of frequently occurring statements within each category. Common emerging codes were grouped to form subcategories.

## Results

**Fig. 1:**
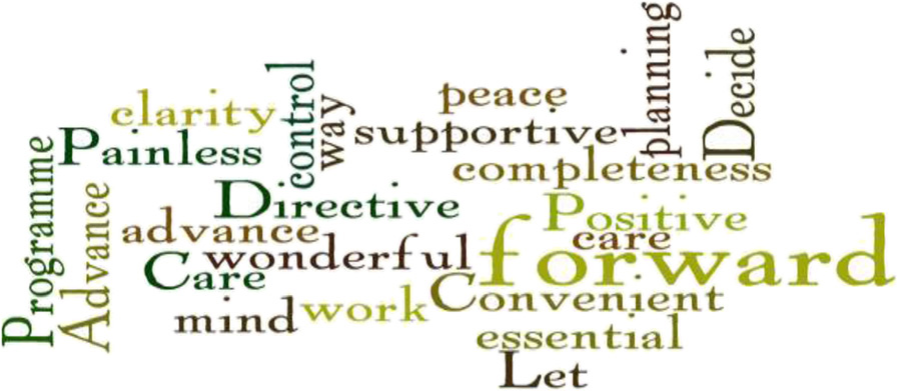
Wordle of descriptors of the ‘Let Me Decide’ programme

The majority of respondents were between the ages of 41–60. Two had a qualification in gerontological nursing and the average length of time working in care of the older adult was 14.5 years. Eleven people had attended accredited education sessions delivered as part of the LMD-ACP programme, others received information/instructions on the programme from formal/informal sessions delivered in the homes throughout the life time of the intervention. Each focus group began with everyone introducing themselves and providing one word to describe the LMD-ACP programme. A wordle was created to reflect these descriptions (Fig. 1).

Words used included: positive, painless, convenient, way forward, supportive, essential, completeness, forward planning, advance care.

In the main focus groups five key categories were pre-established based on the interview guide with 16 corresponding subcategories emerging from the data. These subcategories emerged as a result of 37 codes. See Table 1.

**Table 1:** Categories, subcategories, codes

Categories	Subcategories	Codes
Implementing advance care planning	Directing care	Essential for practice
		Care planning for the future
	Apprehensions and fears evaded by support and usable materials	Fear of unknown
		Support from research team
		User friendly resources
	Emotive process	Emotive process
Benefits	Enhancing communication	Pathway for difficult conversations
		Normalising death
		Building relationships
	Changing care culture	Composed care environment
		Promoting multi-disciplinary awareness
		Enhancing practice and profession
	Avoiding crisis decision making	Reducing emotional distress
		Family preparedness
		Reduce end of life hospital transfer
	Preference-based care	Knowing how to care
		Dignity to decide
Challenges	Establishing capacity	Persons lacking capacity
		Capacity assessment
		Borderline capacity
	Enactment of ACP	Ensuring compliance
		GP involvement
		Legal aspects
	Indecision	Gaining consensus
		Misperceptions of purpose
		Not for everybody
Disadvantages	Resource Intensive	Time and Effort
		Reviewing and updating
Recommendations	Education/training	Train the trainer model
		Blended approach and simulations
	MDT approach	Role of senior nurse and managers
		Getting everyone involved
	Documentation	Capturing conversations
		Sticker alerts on charts
	External support	Link facilitator
		Freely available ACP tool kits
	Introduce concept around admission	Introduce concept around admission

### Category 1: implementing advance care planning

This category relates to the initial reaction to the programme when first introduced and reflections on the process of implementation. Subcategories include ‘directing care’, ‘apprehensions and fears evaded by support and usable materials’ and the ‘emotive process’. Within the subcategory of directing care two codes were identified; essential for practice and care planning for the future. Participants described the significance of the programme in practice:

*“…we are just negligent to look after people without finding out what their wishes are and I think we have no right to look after people without asking them- give them the opportunity.”*

The second subcategory was ‘apprehensions and fears evaded by support and usable materials’, which consisted of three codes; fear of the unknown, support from research team and user friendly resources. For many the process included both positive and negative aspects and some stated that they initially feared getting involved in advance care planning. However support from the research team ensured successful engagement with the programme. According to the participant feedback, the user-friendliness and convenience of the resources, such as patient packs, laminated visual education aids, also made the process of implementation more seamless.

This is highlighted in the following quotes from participants:

*“There was very good support from the research team, without them being intrusive. There were clear lines of communication and we were always informed in advance about when the research team were coming on site and what their visit would involve.”*

*“The other thing is the packs, I love the packs. It’s just so handy to grab the packs. I like the yellow sheet where you list the number of conversations because it shows the evidence of the talks.”*

The final subcategory in this section was ‘emotive process’. Participants felt that dealing with families and the residents themselves was a very emotional process. Many conversations and discussions brought up sensitive issues and some were relieved to have these conversations while others found it difficult to talk about death and dying:

*I have had daughters crying at meetings you know. Because they never knew what their parent wanted and they were reluctant to bring it up with them I think”.*

### Category 2: benefits

Within this category participants spoke about the benefits the programme had brought to their practice, care setting and those they cared for. Four subcategories were identified: enhancing communication, changing care culture, avoiding crisis decision making, preference-based care. Overall the focus group discussions were dominated by the benefits of using the programme.

For enhancing communication three codes emerged from the text. These were focused around ‘pathways for difficult conversations’, ‘normalising death’ and ‘building relationships’. These were seen as key benefits of implementing the programme. One comment from a Clinical Nurse Manager shows how conversations were started as a result of the programme. ‘Let Me Decide’ created a pathway or forum for difficult conversations:

*“…it helped to ease everybody into this conversation, whereas I think without this tool I think we would never have had this conversation between those two parties [family & resident].”*

The topic of death was normalised as a result of the advance care planning process and participants were also surprised how the relationships with the resident and their family developed and strengthened.

*“I think it makes a good relationship with the nurse and everybody. You have talked at those levels, painful levels with family members and the residents, they see you as maybe someone who is closer to them….”*

Not only was communication enhanced, so too was the care culture. For most, they experienced a significant change whereby there was a more composed and calm environment (composed care environment):

*“…that is the pay off the convenience of it at their end of life whether it is weeks or days, it so calm in comparison to before.”*

Further codes in this category include promoting multi-disciplinary awareness and enhancing practice and profession. Nurses felt that the programme had promoted multi-disciplinary awareness of advance care directives and preference-based care. Healthcare assistances, Physiotherapists, General Practitioners (GPs), Emergency Medical Technicians and Occupational Therapists were all aware of the programme and as it became imbedded into practice, these health professionals were actively seeking the residents advance care directives before making decisions regarding their care.

*“Our G.Ps were delighted with it anyway and they keep saying when people are in we have this and we have Advanced Care Directives and everything here in this home and they would be sort of boasting about it that we have it and it is great. They all think it is great.”*

Participants in the focus groups spoke about the many ways the programme has enhanced their practice, how it gave a person-centered, structured approach to ACP and EOL care:

*“I think staff were willing to overcome any difficulties they encountered because they could see the importance of the programme in the longer-term. When they started seeing the benefits of having been through the advance care planning process with residents and families, how it made such a big difference when the resident came to the end-of-life, the staff could see that it was worth all the effort involved.”*

*“I think it has made end-of-life care in general smarter since we started it. I think we have examined critically our end-of-life care”*

The third subcategory within the benefits section highlighted the use of the programme to assist in ‘avoiding crisis decision making’. This was composed of the following codes: ‘reducing emotional distress’, ‘family preparedness’ and ‘reducing end of life hospital transfer’.

Throughout the focus groups participants continually highlighted how crisis decision making was no longer the norm and that family and staff were not under pressure to make last minute decisions.

*“And the family are aware of it. The uncertainty and how we are going to deal with the family and they are all going in different directions - it is all done, there is no anxiety, there is no arguing between families.”*

Families are more prepared at end of life. Respondents felt that families were happy that difficult issues were dealt with in advance. One exemplar demonstrates this:

*“I have received very positive feedback from relatives after their loved one has passed away and some of the feedback directly relates to the level of preparedness of the family and next of kin as a result of LMD. Being prepared and understanding what to expect at this difficult time has helped family members deal with the loss of their loved one.”*

A similar study in Canada using the ‘Let Me Decide’ programme showed that hospital transfer were reduced as residents’ end of life wishes were to remain in the nursing home. This project has also demonstrated these trends. There was agreement among participants, that the number of transfers to hospital resulting in death, had reduced significantly;

*“…we have had a reduction in the number of transfers to acute hospital at the end of life, the staff are happier that they are not seeing dying residents transferred out of their home to a busy A/E Department.”*

The final subcategory in this section was the benefit of being able to provide preference-based care to residents of the homes involved in the project. Staff felt they were now in a position to provide care that was largely based on the wishes of residents. For many this created a sense of ‘knowing how to care’:

*“I think what we were doing before is we were talking about it. When the resident had a turn or became unwell, whereas now we are doing it from you know more or less when they come through the door.”*

Some of the nurses stated that feedback from residents was overwhelming, with many thanking the nurses for promoting autonomy and providing an opportunity to make end of life treatment decisions:

*“I know in a nursing home it is kind of like maybe you relinquish all control of your life and you are running off somebody else’s agenda, it is task orientated. Whereas, you are giving them the dignity of making that decision.”*

It is evident that there are multiple benefits to systematically implementing advance care planning using the ‘Let Me Decide’ programme. One Director of Nursing stated:

*“There is nothing about the programme that I think should be changed. From my perspective, as Director of Nursing, I have not identified any disadvantages to using the LMD programme, only benefits resulting from its implementation”*

### Category 3: challenges

There were lengthy discussions about the challenges of implementation. While some were repeated across focus groups, there was a reassurance from all that although challenges existed, the benefits far outweighed the barriers. Some challenges, in particular focused on ‘establishing capacity’ ‘enacting ACP’ and ‘indecision’ among family and residents.

Establishing capacity was often seen as difficult. This subcategory was made up of codes such as ‘person’s lacking capacity’ ‘capacity assessment’ and ‘borderline capacity’. Staff found it very challenging when a resident was able to clearly articulate certain wishes such as no CPR, but lacked capacity to make decisions around assisted feeding or palliative care versus intensive care.

*“I think there should be a patient’s page for those lacking full capacity to complete an ACD but are clear on some wishes”*

Where this occurred staff documented patient’s wishes and tabled these at designated care planning meeting which involved the patients GP and family. Where possible and medically appropriate the wishes were then incorporated in an ‘end of life decisions care plan’. This enabled the patient’s wishes to be documented despite not having full capacity.

Others found the SIACAD (Screening Instrument to Assess Competency to Complete an Advance Directive) difficult to use initially. While they acknowledged the importance of using a tool to measure understanding of decisions made, some found the terminology problematic for certain residents. The repetitive nature to some of the questions was also identified as an issue:

*“I think the SIACAD is a bit clinical a bit too long you know you are losing their attention.”*

Based on feedback throughout the lifetime of the project, the research team modified the SIACAD. These modifications appeared to make it more user-friendly;

*“Definitely improvements have happened with the SIACAD since we started in the sense that you know we can do a section at a time and they can have the sheet in front of them and they can refer to it. That is a big improvement.”*

Enactment of ACP also proved challenging in certain cases. Examples were given by staff where an ACP was in place, used if the resident was transferred to hospital, however medical staff there were reluctant to adhere to the document, choosing instead to complete a hospital based form which involved discussing the same issues with family again. This was seen as needless and many wished there could be better communication between acute and long-term care.

*“That has happened to one of my residents as well that I sent up with an Advanced Care Directive and it was ignored”*

Other barriers to carrying out an ACP included lack of GP involvement or and statutes to legalise or no national framework for ACDs in Ireland.

A final challenge voiced was issues around indecision. Common codes were ‘gaining consensus’, ‘misperceptions of purpose’ and ACDs ‘not being for everybody’.

“Gaining consensus”, included the sometimes frustrating process of bringing family together and reaching agreement on the course of action. This was not easy at times,

*“And then you would have family members that don’t talk and one member wants this and the other members want that so you have to bring them all together. In a circle where the patient isn’t compos mentis you have to make sure that the whole lot of them are on the same wavelength otherwise you could forget about it.”*

There were a number of residents and family who misinterpreted or had misperceptions about the motive for conducting ACP. Some felt that it was a way to avoid hospital treatment, while others felt ‘God’ would make the decision for them and were averse to the idea of discussing such issues. According to the focus group participants there was a cohort of residents that felt there must be bad news coming if the topic of end-of-life care was being raised. One nurse described the reaction she received from a resident when she introduced the programme:

*“I approached a resident one day about it and he said to me “no I don’t want to have anything to do with that now at all” he said “because I know well what is going on here. They just don’t want the likes of me turning up in hospital”, he said.”*

There was consensus among the focus group participants that ACP was not for everyone. There were challenges inherent in this when a resident would suddenly deteriorate. Providing care to those without ACPs was much more complex and shrouded in uncertainty.

### Category 4: disadvantages

A few disadvantages to using the programme emerged during the interviews. These related to ‘time and effort’ and ‘reviewing and updating’. Staff expressed their concerns about the process of reviewing on an annual basis and were unsure of the level of detail that should be discussed at review. Some feared that residents would become frustrated when it was brought up again and they believed it was complete and a closed subject. Everyone agreed that although a huge amount of time and effort goes into completing the entire process with a resident and their family, it was worth it in the end.

*“I think that once staff began to see the benefits of their hard work delivering ACP to residents and families, they felt that it was worth putting in the effort, even in the face of time constraints”.*

Completion of a full ACD took from two to six hours over a number of meetings/weeks, depending on the resident’s capacity level (as measured by the SMMSE), availability of family, and ward environment.

*“You need quite a few meetings actually, you have to introduce it, introduce the literature and then try and get the relatives and give them the literature and explain it all to them…and well of course you have to do the SMMSE first you know so it starts there really. I suppose it does need that amount of consideration it is a very big decision.”*

### Category 5: programme recommendations

A number of recommendations were suggested by the focus group participants. They ranged from methods of providing education and training, to timing of introducing ACP to residents and their family. Within this category there were five subcategories; education/training, MDT approach, documentation, external support, and introducing the concept around admission.

Recommendations on education and training were focused on the type of delivery. There was consensus among the groups that the face-to-face sessions were excellent as they facilitated meaningful discussions around this difficult topic. While they agreed that the use of online education for defined components of the training would alleviate the pressure of trying to attend sessions, they recommended a blended approach, i.e. use of on-line and face-to-face.

*“I thought the face-to-face educational sessions were extremely useful for the staff to be able to discuss these EOL issues openly with the support and understanding of your work colleagues – this brings a richness to the educational experience. I would worry about how staff would engage with an online educational programme – it would not offer the same incentives or richness as the group training sessions.”*

Simulations or demonstrative-type sessions were also suggested, whereby the whole process was completed using role-modelling and case scenarios. For a larger scale roll- out, the nurses felt that a train-the-trainer model would be most beneficial.

Nurses also recommended a multi-disciplinary approach from the start of the process. This encompassed getting everyone involved including, healthcare assistants, GPs etc.

*“Because a lot of times when you go around you find that the residents will talk more with the health carers, the person washing the floor they will talk more with than they talk to the person coming into care for them and wash them.”*

It was apparent from the regular visits to the nursing homes that the role of completing an ACD was undertaken primarily by senior staff (Clinical Nurse Managers). During the focus group sessions this observation was confirmed. Most felt that it was such an important aspect of the person care planning that the core aspects of it needed to be led by senior staff. One Director of Nursing clearly articulates the rationale for including it as part of a senior nursing role;

*“I would see the delivery of advance care planning as a role for senior nursing staff/ward managers –at this point in time anyway, at least until the process is established and clinical staff have the required competencies to engage in the process. The senior nursing staff are usually the people that the family come to anyway. They have the status and the families have trust in them. It is a highly difficult and sensitive area and you need to be competent and very confident to have these discussions with residents and families.”*

Documentation of conversations was also recommended and these were described as learning tools that staff reflected on when trying to improve their communication skills around the care planning process.

*“I found taking notes worked really well and when I look back on the early ones now I cringe because just the wording you are almost leading them whereas now I have a much better approach into it. What I say to everybody is you know would you like to talk about what you like to do if you became sick.”*

Once conversations were captured and the ACP process completed staff recommended placing sticker alerts on the charts so that everyone was aware and could easily access the content of the plan in case of an emergency situation.

*“We put a sticker on the outside of the charts so even at a glance before you can pull everything off the shelf you see the sticker and you know that there is an ACD”.*

The final two recommendations related to external support i.e. having a link facilitator to liaise with if problems arose, having freely available tool kits, and introducing the concept around time of admission. On admission appeared to be the most opportune time to talk about the availability of this care option in the nursing home.

Overall, it is evident from the focus groups that the programme was evaluated in a very positive light. All the homes have now embedded the programme as part of their care packages. One Director of Nursing stated that she felt staff morale had improved as a result of the programme. It was unanimous that the programme should be implemented in other care homes and all agreed that it was now a core aspect of their practice, which they would not ever do without again.

## Discussion

The systematic implementation of an advance care planning and palliative care education programme in long-term care is a novel approach to facilitating self-determination among residents, build capacity for delivering palliative care among staff, and improving the quality of end-of-life care. The ‘Let Me Decide’ programme was designed to encompass and address these key issues when caring for older adults. A qualitative evaluation of the programme by means of focus groups with key informants allowed for the identification of specific benefits of implementing this programme and also provided a platform to highlight challenges and recommendations. Benefits of the programme, according to participants, ranged from reducing crisis decision making among family members to providing pathways for difficult conversations. Crisis decision making can lead to feelings of regret among family members and subsequent depression. The reduction of post-traumatic stress disorder and depression among family members as a result of engaging with advance care planning is well documented [1].

Inextricably linked to crisis decision making is the reduction of end-of-life hospital transfers. ACP conducted in a systematic way reduced hospital transfers [4, 12]. Findings from this qualitative evaluation demonstrated that staff noted a marked reduction in hospital transfers which was consistent with the wishes of residents who had completed the ACP process. Inadequate planning and poor communication are antecedents to hospital transfer [13, 14]. It is important that programmes such as ‘Let Me Decide’ are promoted and implemented to facilitate conversations and planning for end of life. Other emerging factors such as the patient’s medical condition and ability of the home to manage their care also impact on the decision to transfer out. In certain instances transfer is appropriate and should not be seen as a failure of care or reflect the quality of care provided. A recent systematic review found that up to 2.5 % of hospital admissions were from long-term care with an estimated 30 transfers for every 100 residents. In the majority of cases, falls or musculoskeletal problems were cited as the reason for referral [15]. Thus, many hospital transfers are appropriate and necessary.

Some staff felt that the programme had ‘stilled the waters’ at end of life and created a composed care environment. There is evidence from this study that families felt more prepared for the death of their loved one by engaging with the programme and had reduced feelings of stress and anxiety. Feedback from family to staff suggested that they were able to find solace in the fact that the death had occurred in accordance with the wishes of the resident.

From the interviews it was clear that a shift in culture had occurred. Many stated that dying and death was no longer a ‘taboo’ subject, rather a normal part of the care environment. Normalising death as part of the life process may help to reduce emotional distress among staff [16] and other residents within the home. Indeed participants reported that many residents felt a great sense of relief when the topic of end-of-life care was raised. There is consensus in the literature that residents in long-term care, in general, are comfortable engaging in conversations around dying and death [17].

Significant barriers to ACP in long-term care included establishing resident’s capacity to complete an advance care plan or directive. This may create difficulty for staff as some of the residents may have capacity to make less complex decisions about their care i.e. not to transfer to hospital. However, issues such as level of care (palliative, limited, surgical, intensive) desired, if a life-threatening illness occurred may not be fully understood. Determining capacity in ACP is a dominant challenge [18, 19] particularly in light of the growing numbers of residents in long term care with cognitive impairment. To reduce this burden it is necessary to begin targeting community based people with a structured ACD process, using standardized forms [20]. For example, GPs or community programmes could offer education and facilitate completion of ACDs to healthy older adults or at point of chronic illness diagnosis, such as Parkinson’s disease or dementia. This would ensure, eventually, that those admitted to long-term care would have completed ACDs before they became cognitively impaired.

The timing of introducing ACP was discussed during the focus groups. There was consensus among the groups that on admission family, and residents should be made aware that ACP is part of the home’s patient-centred approach to care. For new residents admitted to the home, staff have recommended an amendment to their policy which states that the concept of end of life care planning be introduced to family and residents at time of admission. This finding is echoed in similar research [17, 18]. Knowing when to have the conversation can be difficult especially in community based settings such as GP practices [21]. However in long-term care, owing to the 24/7 nature of care delivery, nurses become very familiar with families and residents and are more sensitive to recognise the ‘right moment’ to introduce the subject. In the current study this ‘moment’ was usually prompted by a resident who would refer causally or formally to an end of life issue which could be self-involving or regarding a member of their community. As the programme commenced at one point in time there may have been residents living in the nursing home for a number of years and therefore timing the ‘right moment’ was essential to starting the conversation.

Recommendations for use of the programme, or similar ACP initiatives included the need to involve all members of the multi-disciplinary team. GPs play a particularly important role in the process and are often the key decision makers when challenging clinical scenarios arise. When the GP is clear about the families’ and residents’ wishes, decision making is less complex. However, if there is conflict or uncertainty, issues such as fear of litigation [13] can dictate place of death and course of treatment [19].

Involving junior staff in the process can also improve the team’s approach. In most of the homes that participated in this study, senior nurses took ownership over the role. However, they admitted that sometimes the more junior staff, care assistants and even household (cleaning/catering staff) have meaningful conversations with residents that could inform the ACP process. It is essential that all members of the care team are educated on preference-based care and can recognise moments of meaningful conversations with residents. Staff education in advance care planning is essential [18] and the education sessions provided as part of the ‘Let Me Decide’ programme were well received. The combined ACP and palliative care education programmes were identified as a critical hybrid. Most residents requested palliative care when faced with a life-treating irreversible condition. This meant that staff needed to be skilled in a palliative care approach. Most felt strongly that future delivery of education sessions should include an online and face-face simulation type workshops. Staff liked the idea of role playing and suggested that this improved confidence when completing an ACD with a resident. A blended approach to learning has gained increasing support over the years [22] and is encouraged when delivering education on ACP [17].

While the merits of systematically introducing ACP into long-term care are apparent, the process itself is time-consuming and resource intensive for staff that are willing to take on the role [19]. These issues were raised by those who participated in the focus groups. Many felt in particular, that the first few ACPs took a lot of time. However as with any skill, over time they felt more confident educating residents and their families and were even more assured that it was worth the effort when a resident with an ACD/ACP died in accordance with their stated wishes.

## Conclusions

The programme has been well received by staff. Feedback from the focus groups indicates that all the homes have now embedded the programme as part of their care packages. Many have stated that the programme has transcended a number of care issues in the home and is much more than just a directive. Participants indicated that relationships with residents had deepened and there is more open and honest communication with family. End-of-life care is now focused on symptom management, comfort and addressing spiritual care needs as opposed to crisis decision making and family conflict. Staff morale has improved as a result of the programme. Participants were unanimous in the belief that the programme should be rolled out to other care homes and all agreed that in the future, they would not want to practice as healthcare professionals without it. The programme was unequivocally identified as an essential component of practice in long-term care and recommended for extensive implementation at local and national level.

## Abbreviations

ACD: Advance Care Directive
ACP: Advance Care Planning/Advance Care Plan
CPR: Cardio-pulmonary resuscitation
EOL: End-of-Life
GP: General Practitioner
LMD: ‘Let Me Decide’
LMD-ACD: ‘Let Me Decide’ Advance Care Directive
LMD-ACP: ‘Let Me Decide’ Advance Care Planning
LTC: Long-term care
RCT: Randomised Control Trial
SIACAD: Screening Instrument to Assess Competency to Complete an Advance Care Directive
SMMSE: Standardised Mini-Mental State Examination
